# Delayed management of atrial lead dislodgment after pacemaker implantation: a case report

**DOI:** 10.1186/s13256-020-02626-z

**Published:** 2021-01-14

**Authors:** Fu Guan, Guangping Li, Yong Liu, Xing Gao, Rui Zhou

**Affiliations:** 1 Department of Cardiology, Capital Medical University affiliated Beijing Shijitan Hospital, No 10 Tieyi Road, Haidian, Yangfangdian, Beijing, China; 2 Department of Echocardiogram, Capital Medical University affiliated Beijing Shijitan Hospital, Beijing, China

**Keywords:** Lead dislodgement, Case report, Pacemaker, Complication

## Abstract

**Background:**

Pacemaker lead dislodgement may cause malfunction in the pacing system, which may lead to severe adverse events. For patients with sick sinus syndrome but normal atrioventricular conduction, atrial lead dislocation may cause excessive unnecessary ventricular pacing, resulting in nonphysiological pacing leading to heart failure. The longer the unwanted ventricular pacing continues, the greater the chances that irreversible heart failure may occur. Ironically, we admitted a patient who had been refusing dislodged lead relocation for 7 years. The symptoms of heart failure were significantly resolved after new atrial lead implantation. We reviewed her clinical data before and after the procedure and believed the case was worthy of reflection.

**Case presentation:**

An 83-year-old Han Chinese woman presented with heart failure symptoms for 7 years due to the late macro-dislodgement of an atrial pacing lead. Her echocardiogram showed average left ventricular ejection fraction (LVEF) but reduced left ventricular end-diastolic volume (LVEDV) during right ventricular pacing, indicating heart failure with preserved ejection fraction (HFpEF). After 7 years of refusal, she finally agreed to implantation of a new atrial lead. She has been doing well since the operation.

**Conclusions:**

For patients with sick sinus syndrome with dual-chamber pacemaker indication, atrial lead dislodgement should be appropriately managed if the atrioventricular function is normal. As the consequences are subtle and appear gradually, they might be overlooked by patients and even doctors. Implanting a new atrial lead is the right thing to do rather than just passively waiting or treating with symptom relief medications.

## Background

Pacemaker lead dislodgement causes malfunctions in the pacing system. Late dislodgement is defined as dislodgement occurring more than 6 weeks after pacing system implantation [1]. Classification of lead dislodgement includes macro-dislodgement and micro-dislodgement. Micro-dislodgement refers to minor dislocation of the pacing lead that cannot be identified through radiography, while macro-dislodgement can be observed directly from radiography. For patients with sick sinus syndrome but normal atrioventricular (AV) conduction, atrial lead dislocation may cause excessive unnecessary ventricular pacing, resulting in nonphysiological pacing leading to heart failure. Furthermore, as its clinical manifestations are usually subtle during the first several months, patients may overlook this problem. Here we report a case of an 83-year-old Han Chinese woman who experienced symptoms of heart failure for over 7 years due to late atrial lead macro-dislodgement after pacemaker insertion. Her experience through the 7 years is worthy of our attention.

## Case presentation

The patient was an 83-year-old Han Chinese woman with recurrent dyspnea for over 7 years. She had undergone dual-chamber pacemaker insertion for sinus bradycardia and sinus arrest 8 years earlier. Echocardiography showed normal left ventricular function before the procedure. No perioperative complications occurred. She had no unpleasant complaints after the procedure. At a 3-month routine follow-up of pacemaker interrogation, atrial lead dislodgement was detected. Further radiography confirmed atrial lead macro-dislodgement. As a result, an immediate atrial lead relocation was recommended by the doctor. However, she insisted that she felt no discomfort and refused any therapy or medication. One year later, she was admitted to the hospital for exertional dyspnea. Subsequent echocardiography revealed normal left ventricular ejection fraction (LVEF) and mild mitral valve regurgitation, similar to the findings before pacemaker implantation. Computed tomography coronary angiography revealed no signs of coronary artery stenosis. Pacemaker interrogation revealed 50% VVI pacing. Mild elevation of serum N-terminal prohormone brain-type natriuretic peptide (NT-proBNP) with a value of 900 pg/ml (normal range 0–300 pg/ml) was detected. Since she still refused lead reset, she was treated with diuretics and discharged from the hospital after symptoms improved. The discharge diagnosis was atrial lead dislodgement and heart failure. However, over the past 7 years, the patient experienced no aggravated heart failure symptoms, but never discontinued diuretics.

**Fig. 1:**
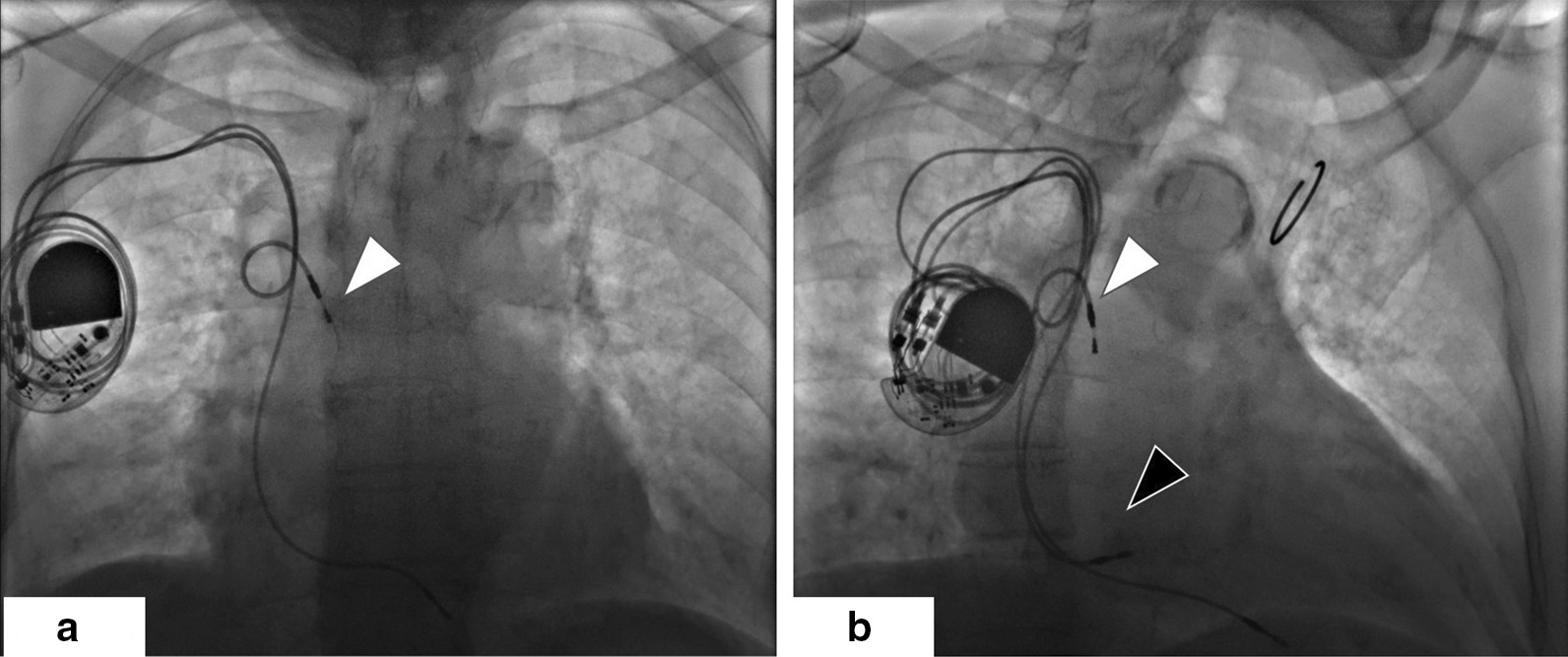
Anteroposterior projected radiograph of the patient before (**a**) and after (**b**) new atrial lead implantation. **a** The dislodged atrial lead in the superior vena cava, indicated by the white arrow. **b** The new atrial lead implanted in the right lower septum, indicated by the black arrow

**Fig. 2:**
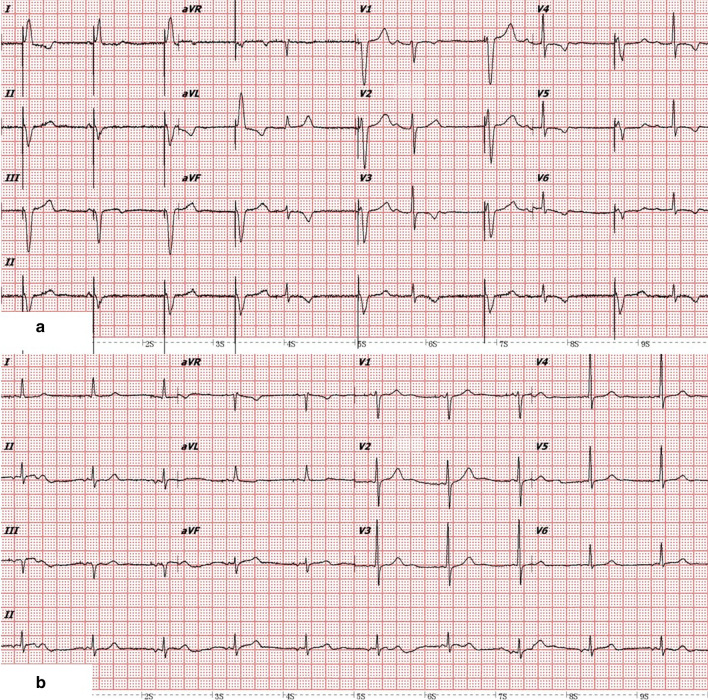
Electrocardiogram before and after new atrial lead implantation. **a** Before new atrial lead implantation, VVI pacing showed a large percent of ventricular pacing (1st–4th, 6th, 8th, 10th QRS) with less atrial deflection followed by intrinsic ventricular deflection (5th, 7th, and 9th–11th QRS). **b** After new atrial lead implantation, each atrial deflection was followed by intrinsic ventricular deflection, shown as AAI pacing mode

**Fig. 3:**
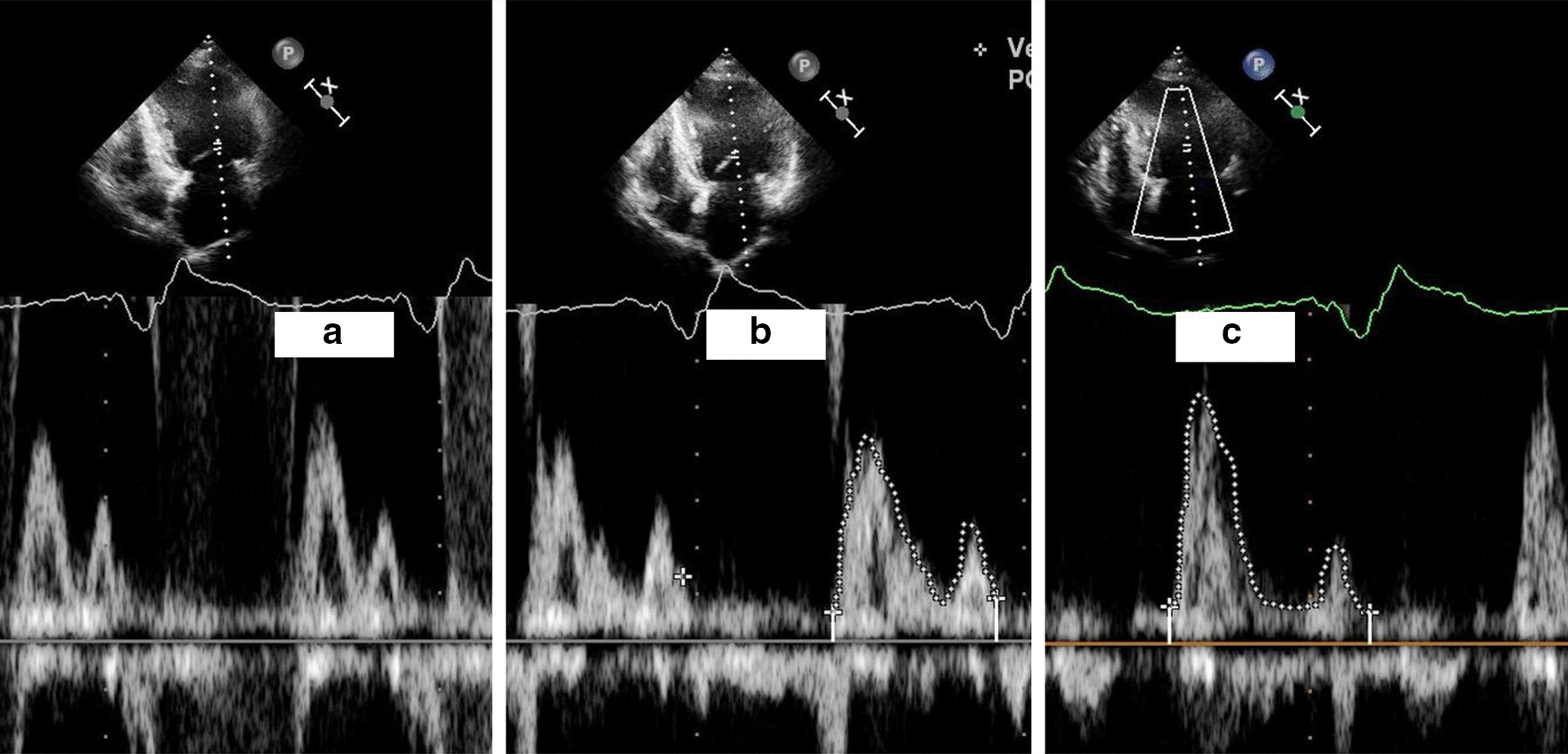
Mitral Doppler recordings. **a** EA fusion during ventricular pacing before new atrial lead implantation. **b** *E* wave and *A* wave were separated only during a few intrinsic ventricular deflections before new atrial lead implantation. **c** *E* and *A* waves were separated ideally during DDD pacing mode, with atrial deflection followed by intrinsic ventricular deflection conducted through the normal AV node

At the latest admission in March 2019, she had severe dyspnea even with mild physical activity. Physical examination showed that body temperature was 36.2 °C, pulse 66 beats/minute, irregular; blood pressure was 105/80 mmHg; the respiratory rate was 22/minute, and oxygen saturation was 98% on room air. Jugular venous distension was not obvious. Cardiac examination revealed irregular rhythm, no murmur or rub. Lung auscultation revealed rales at both lower sides. Chest X-ray showed right atrium lead dislodgement with the distal part located at the inferior segment of the superior vena cava. In contrast, the right ventricular lead appeared to be normally positioned in the right ventricular apex (Fig. 1a). Electrocardiogram (ECG) showed VVI pacing (Fig. 2a). Echocardiogram revealed no wall motion abnormality, with normal LVEF of 66%, mild mitral valve regurgitation, and tricuspid valve regurgitation with mild pulmonary hypertension. Device interrogation revealed VVI mode with 80% right ventricular (RV) pacing and normal RV lead parameters. The generator longevity was 4 to 5.5 years. Serum NT-proBNP was at a higher level of 4200 ng/ml. Notably, further echocardiogram during the irregular rhythm showed significantly reduced left ventricular end-diastolic volume (LVEDV, 70 ml/m^2^) and pseudonormal LVEF (68%) during ventricular pacing, compared with LVEDV of 88 ml/m^2^ during intrinsic ventricular deflection. Moreover, we found *E*/*A* wave fusion with insufficient diastolic filling during ventricular pacing (Fig. 3a, b). These supported the diagnosis of heart failure with preserved ejection fraction (HFpEF) due to AV desynchronization. After the third consultation with the patient, informed consent was provided for new atrial lead implantation. However, because of a health insurance problem, she did not approve simultaneous extraction of the dislodged lead. A new active fixation atrial lead was fixed in the right lower septum to achieve reliable atrial pacing. The device was then reprogrammed to a dual-chamber system, and interrogation revealed a DDD pacing mode with atrial pacing followed by intrinsic ventricular rhythm (Fig. 1b). The procedure was uneventful. During 6 months of follow-up, she was doing quite well with the cessation of diuretics. The follow-up echocardiogram showed sufficient AV synchrony with normal LVEDV and LVEF (Fig. 3C). No pulmonary hypertension or cardiac effusion was found.

## Discussion and conclusions

Atrial lead macro-dislodgement after dual-chamber pacemaker implantation is symptomatically subtle initially but potentially dangerous in the long run. Early atrial dislodgement happens in 3.8% of patients with DDD implantation. Moreover, it is one of the most common reasons for re-intervention after pacemaker implantation. Within the first several weeks of pacemaker implantation, the actual cause for lead dislodgement is difficult to trace. According to the current literature, twiddler's syndrome and reel syndrome are the most commonly identified causes of lead dislodgement [2, 3]. When patients manipulate the pulse generator inadvertently or deliberately, these syndromes may happen. When the generator is turned and rotated on its long axis, twiddler's syndrome would occur if the lead rolled around the generator and caused lead dislodgement. In contrast, when the generator is turned and rotated on its transverse axis, reel syndrome would occur if the lead rolled around the generator and resulted in lead dislodgement. The patient in our case had no such experience of generator manipulation. However, consistent with the predisposing factors for the above syndromes, she was indeed an aged obese woman. Thus the generator pocket gradually expanded due to flabby subcutaneous tissue, which would allow for generator displacement inside the pocket. Other causes, including direct trauma over the pacing system [4] and intense respiratory therapy, have been reported as well [5]. These might cause micro-dislodgement rather than macro-dislodgement of the pacing leads, and thus were ruled out according to our patient's medical history.

Clinical symptoms of atrial lead dislodgement depend on the location of the lead and the patient's reliance on the pacemaker. The most common symptom may be discomfort or palpitation due to right phrenic nerve stimulation or excessive atrial pacing. Clinical signs of atrial lead dislodgement include abnormal findings in ECG and device interrogation. Loss of capture, inadequate sensing, and impedance abnormality might suggest lead displacement. However, the final diagnosis of macro-dislodgement of the atrial lead can only be established by radiography. As in this case, the clinical investigations at the previous and latest admission both confirmed right atrial macro-dislodgement.

The management for lead dislodgement depends on the time of pacemaker implantation. Other factors should also be considered, such as the patient's clinical condition, fixation type of the dislodged lead, and working status of the generator and other pacing leads. The present patient missed the chance to reset the dislodged lead initially. Atrial lead reposition would have been straightforward at the very beginning, since there was no endocardial fibrous formation around the distal end of the lead or subclavian vein adhesion of the proximal segment of the lead. In this case, the solution was to implant a new active fixation atrial lead through proximal subclavian vein access.

Before the procedure, we prepared the contralateral subclavian vein as an alternative to introducing the lead via a subcutaneous tunnel in the event of failed ipsilateral venous access. Fortunately, the venogram showed available ipsilateral access, and the new atrial lead was implanted smoothly (about 15 minutes). Furthermore, to prevent possible recurrence of lead dislodgement, the generator must be placed beneath the fascia of the pectoral muscle and sutured with the muscle. This procedure was uneventful, and the patient was discharged the next day. Although nonabsorbable antimicrobial pouches were suggested in a previous study to avoid the recurrence of lead dislodgement [6], we have had no such experience to date.

Moreover, as this patient's AV node functioned well, ventricular pacing was not required initially. Over the 7 years after lead dislodgement, ventricular pacing of up to 80% was revealed due to sinus bradycardia or sinus arrest. Echocardiogram showed LVEDV reduction with pseudonormal LVEF during ventricular pacing. Doppler revealed *E*/*A* wave fusion during ventricular pacing rather than during intrinsic ventricular deflection. These features indicated that ventricular pacing was responsible for AV desynchronization leading to heart failure. It was HFpEF caused by impaired ventricular diastolic filling [7]. To our knowledge, the echocardiogram showed that the traditional pacemaker syndrome had apparent valve regurgitation [8]. However, severe valve regurgitation was not found in our case. After the newly implanted atrial lead restored AV synchrony, ventricular diastolic filling was resumed with a significant splitting of the *E*/*A* wave in Doppler (Fig. 3c). Although the current consensus recommends minimizing right ventricular pacing in patients with sinus node disease but normal AV node function [9, 10], appropriate management of dislodged atrial lead in this population is still controversial. Some experts recommend dislodged lead removal in such cases. However, others recommend no removal of the dislodged lead if there are no related clinical symptoms. Since the distal part of the dislodged lead was located at the inferior segment of the superior vena cava, and no stimulated symptoms existed, dislodged lead extraction remained debatable in this case.

In conclusion, the management of atrial lead dislodgement must be decisive. Postoperative follow-up for cardiac pacing system should be carried out regularly to rule out any possible complications. In this case, implanting a new atrial lead is the right thing to do.

## Data Availability

All data regarding this case report are included in this published manuscript.

## Abbreviations

LVEF: Left ventricular ejection fraction
LVEDV: Left ventricular end-diastolic volume
HFpEF: Heart failure with preserved ejection fraction
ECG: Electrocardiogram
